# Gorham–Stout syndrome, the challenge in diagnosis and unique in treatment: a case report

**DOI:** 10.1186/s13256-023-04094-7

**Published:** 2023-08-22

**Authors:** Sadegh Saberi, Nima Bagheri, Seyyed Hadi Kalantar, Hana Saffar, Seyyed Saeed Khabiri

**Affiliations:** 1 Department of Orthopedic Surgery, Joint Reconstruction Research Center, Imam Khomeini Hospital Complex, Tehran University of Medical Sciences, Keshavarz Boulevard, Tehran, 1419733141 Iran; 2 Department of Pathology, Cancer Institute, Imam Khomeini Hospital Complex, Tehran University of Medical Sciences, Tehran, Iran

**Keywords:** Gorham-Stout syndrome, Bone resorption, Endoprosthesis, Total femur replacement

## Abstract

**Background:**

Gorham–Stout disease is a rare condition with fewer than 400 reported cases in the literature. The presentation of Gorham–Stout disease varies on the basis of location, extent, fracture, and accompanying symptoms. It lacks a specific histopathological appearance but is characterized by vascular changes and the absence of cellular atypia.

**Case presentation:**

This article presents a case study of a 16-year-old Persian boy with an entire femur with Gorham–Stout disease, highlighting the difficulties in managing this condition. The lack of a clear diagnosis resulted in prolonged procedures, delayed medical treatments, and ultimately required total femoral replacement with an endoprosthesis.

**Conclusion:**

It is important to note that raising awareness of this disease and its potential complications can facilitate timely and appropriate treatment for patients presenting in the early stages of the disease.

## Background

Gorham–Stout disease (GSD) is a rare condition characterized by progressive osteolysis and replacement of bone tissue with fibrous tissue. Although the condition was first referenced in a medical article by Jackson in 1883 [1] as “boneless arm,” it was not until 1955 that Gorham and Stout reported the cases of the disease with evident hemangiomatosis [2]. GSD is also known as vanishing and phantom bone diseases and is classified as type IV osteolysis in the Hardegger classification [3].

The presentation and extent of symptoms of GSD are highly variable and depend on the location and extent of the osteolytic process. Given the rarity of the disease and its diverse manifestations, accurate diagnosis can be challenging. In cases of unexplained osteolysis or where bone destruction is extensive, with absent cellular atypia on pathology with evidence of angiomatous proliferation [4], GSD should be considered as a potential diagnosis and referred to a specialized tumor and reconstruction referral center for further investigation and appropriate management.

This case report aims to present a challenging case of GSD, highlighting the difficulties in diagnosis and treatment. The patient’s case involved total femoral replacement surgery, a unique and rare course of treatment for this condition. Through this report, we hope to raise awareness of the disease and its potential complications, as well as to contribute to the knowledge and understanding of GSD.

## Case presentation

**Fig. 1:**
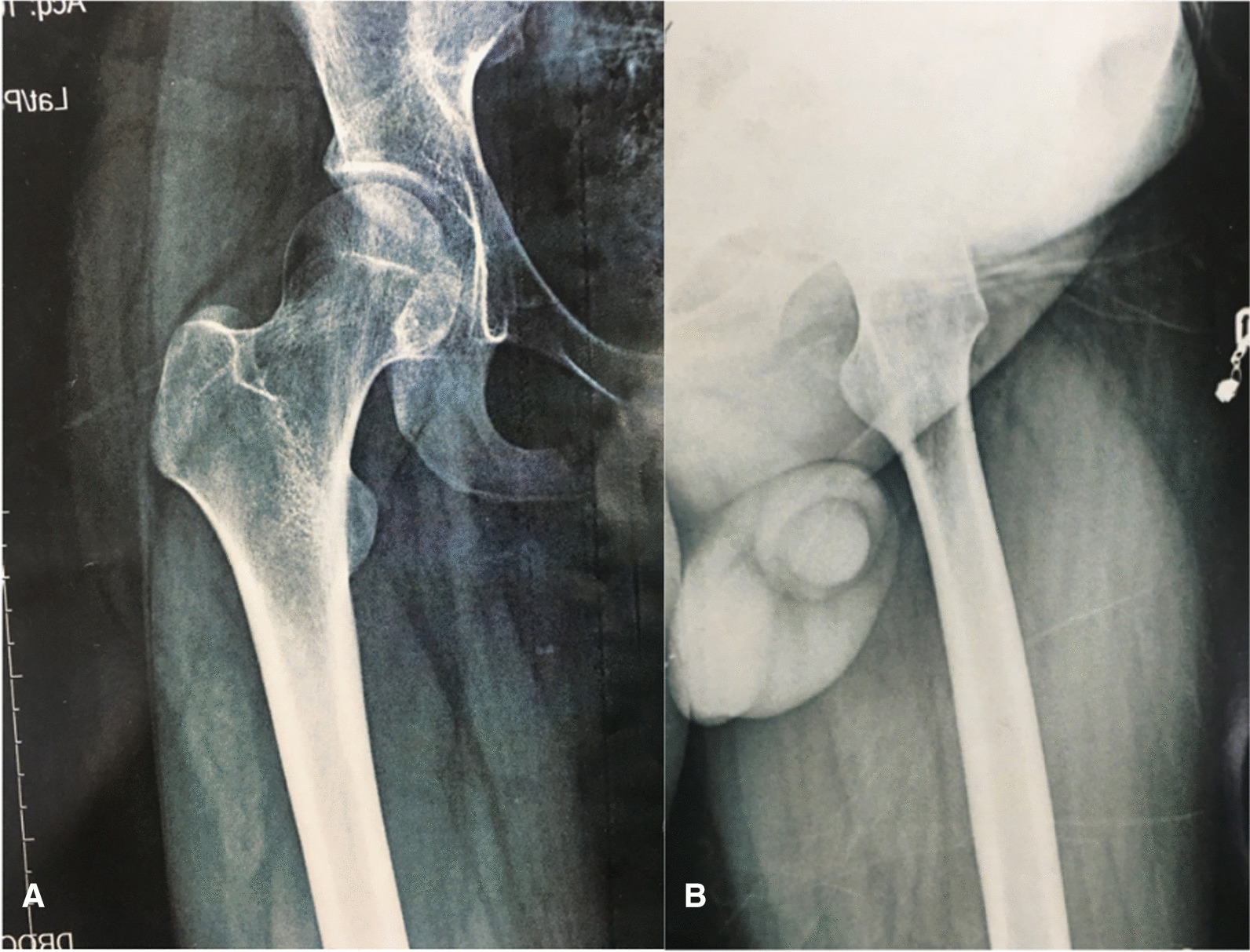
Radiographs of the right hip at initial presentation, including anteroposterior (**A**) and lateral (**B**)

The subject of this case study is a 16-year-old healthy Persian male patient without any past medical history who was referred for right hip pain that had been present for approximately 3 months. The pain initially appeared during physical activity and gradually became persistent. The pain was moderate in intensity and led to limited performance and limping (Fig. 1).

Magnetic resonance imaging (MRI) was performed on the patient. Given the patient’s pain and the existence of a lesion in their proximal femur, along with signal changes detected in their MRI, it was recommended that an initial biopsy be conducted for accurate diagnosis and effective management. The pathology report of the proximal femur specimen biopsy showed “fibromuscular and bony tissue involved by a neoplasm composed of large cystically dilated vessels with thin walls.” Finally, the diagnosis was reported as “cavernous hemangioma of bone.” The laboratory blood test was: calcium 2.48 mmol/L (normal range 2.2–2.7 mmol/L); phosphate 1.15 mmol/L (1.12–1.45 mmol/L); albumin 47 g/L (34–54 g/L); 25-OH-D3 level 52 mmol/L (52–72 nmol/L); C-reactive protein 2 mg/L (8–10 mg/L); erythrocyte sedimentation rate 3 mm/hour (0–15 mm/hour); alkaline phosphatase = 75 unit/L (44–147 unit/L) and revealed normal bone metabolism.

**Fig. 2:**
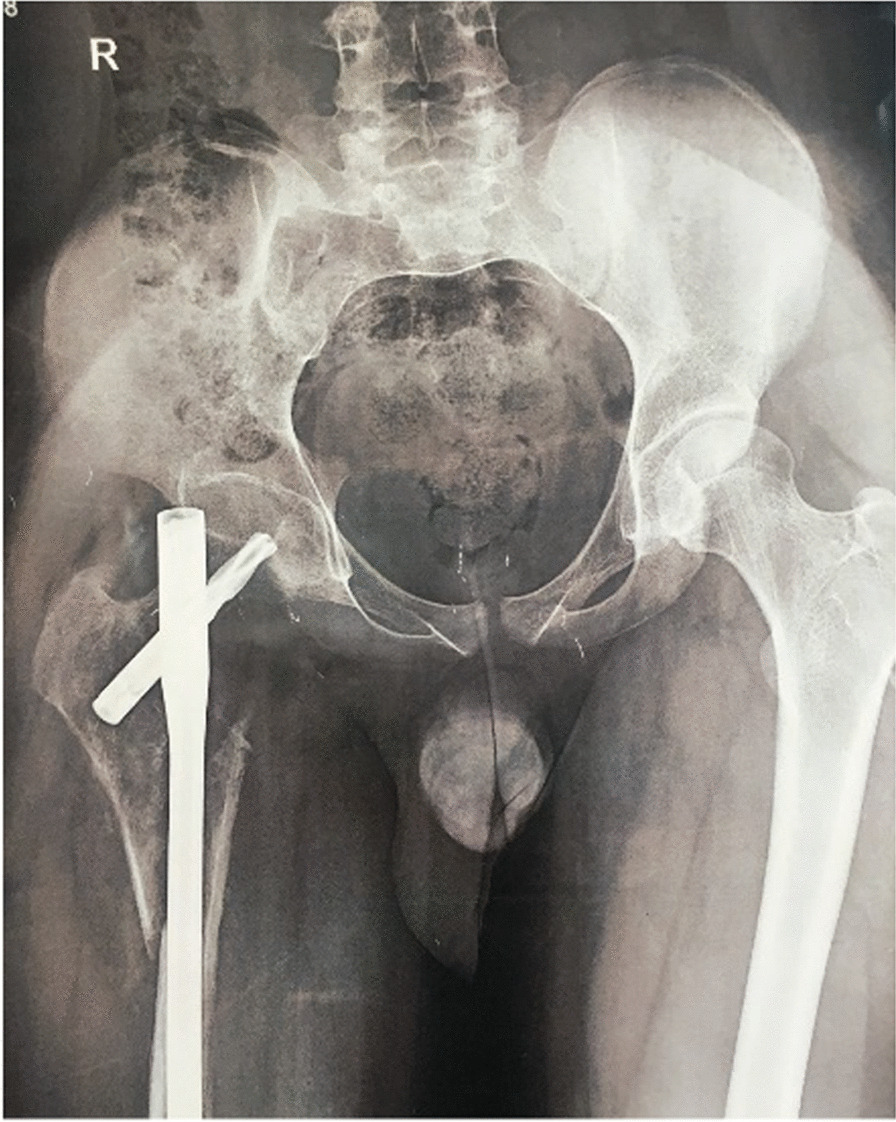
X-ray image showing the extensive lysis of the right trochanteric region and the femoral neck

Due to the patient’s pain, lack of evidence of primary bone sarcoma, and the extent of the lesion, prophylactic fixation with a cephalomedullary nail was performed on the patient. Following the surgery, the patient continued to experience pain and presented with severe pain at the hospital emergency room 1 month after the operation (Fig. 2).

**Fig. 3:**
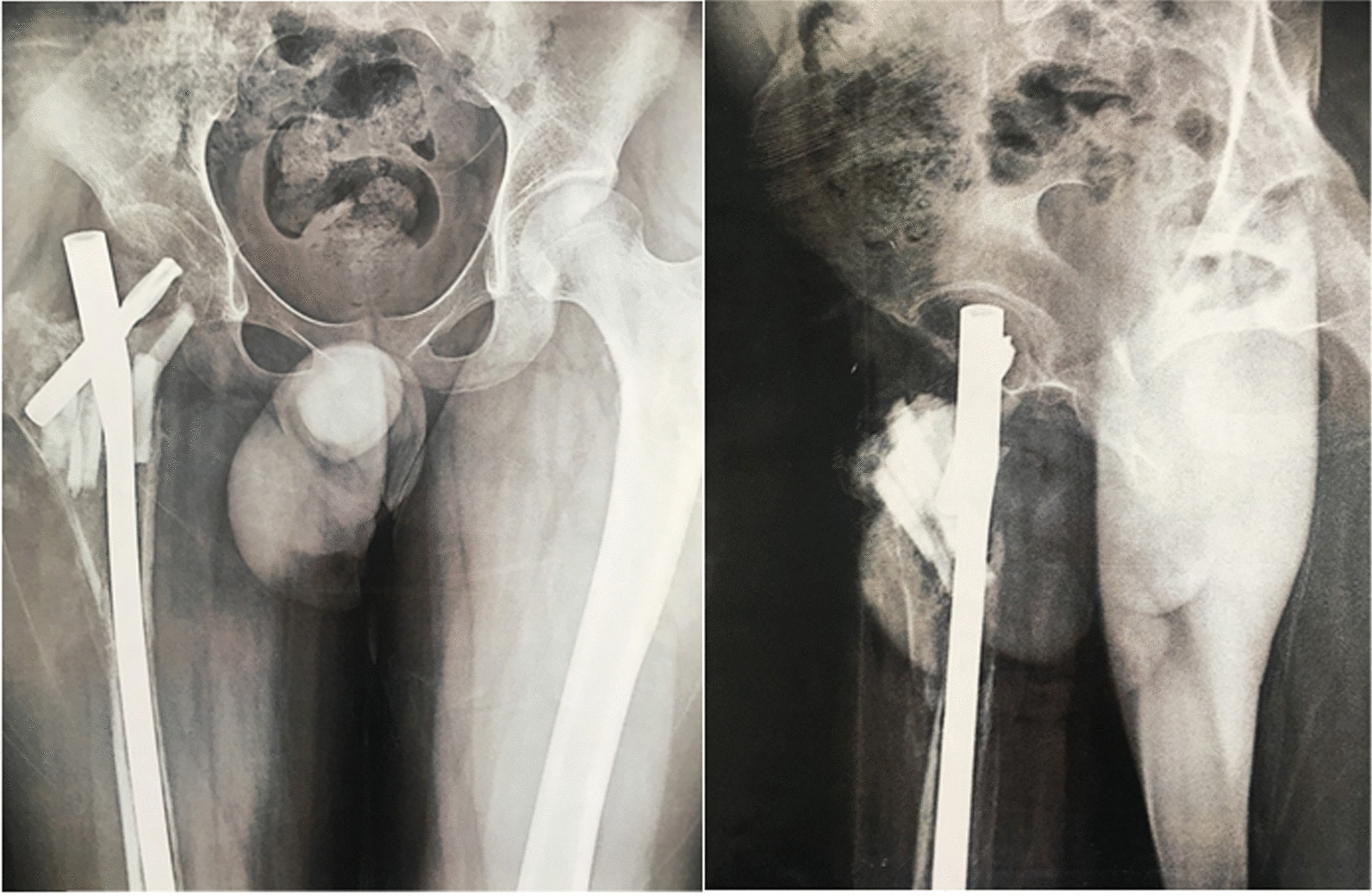
Radiographs of the right hip after revision surgery; note proper reconstruction of the intertrochanteric and femoral neck region by utilizing both structural allograft and cephalomedullary nail

An X-ray revealed a fracture and bone destruction, necessitating curettage, structural fibula allograft, and fixation with a cephalomedullary nail (Fig. 3). There was no evidence of infection or positive specimen culture.

**Fig. 4:**
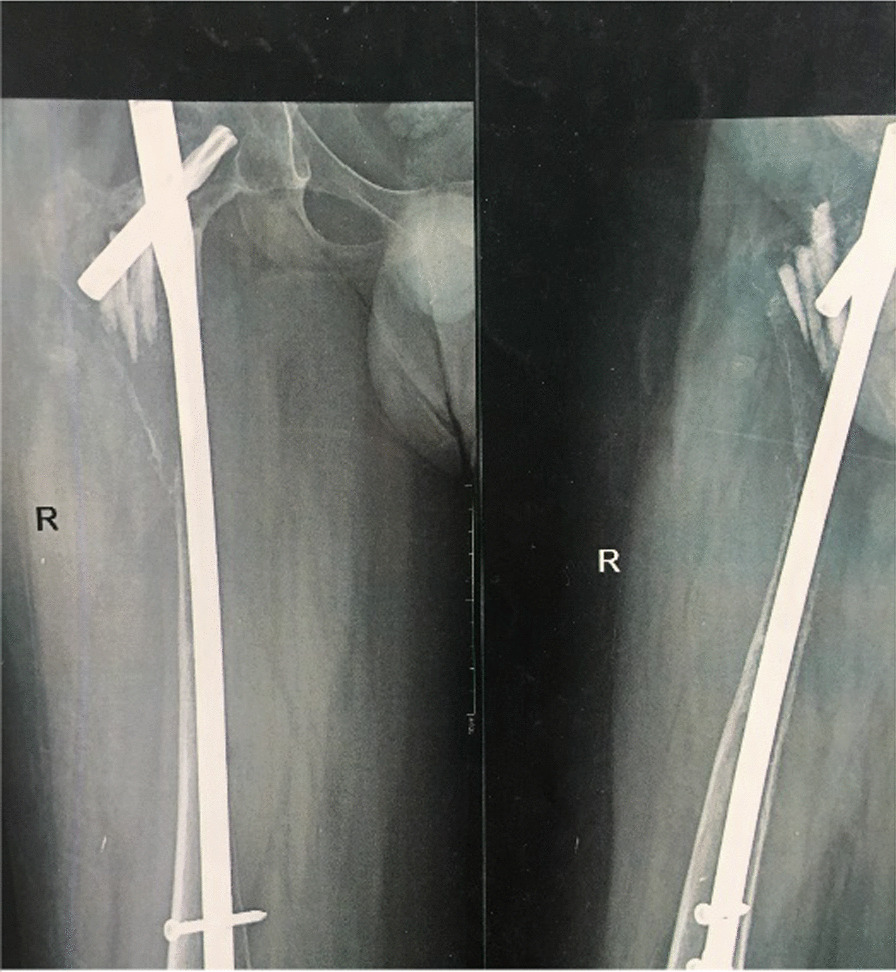
Right femur Anterior Posterior (AP) and lateral X-ray; note allograft resorption and bone disappearing

At the patient’s next follow-up appointment, which occurred 2.5 months later, a decrease in bone density in the proximal femur and absorption of the allograft was observed (Fig. 4). As a result of this osteolysis progression, a second incisional biopsy was performed on the patient. The pathology report showed “cavernous hemangioma and negative for malignancy.” On the basis of this diagnosis, the patient was deemed eligible for sclerotherapy treatment.

**Fig. 5:**
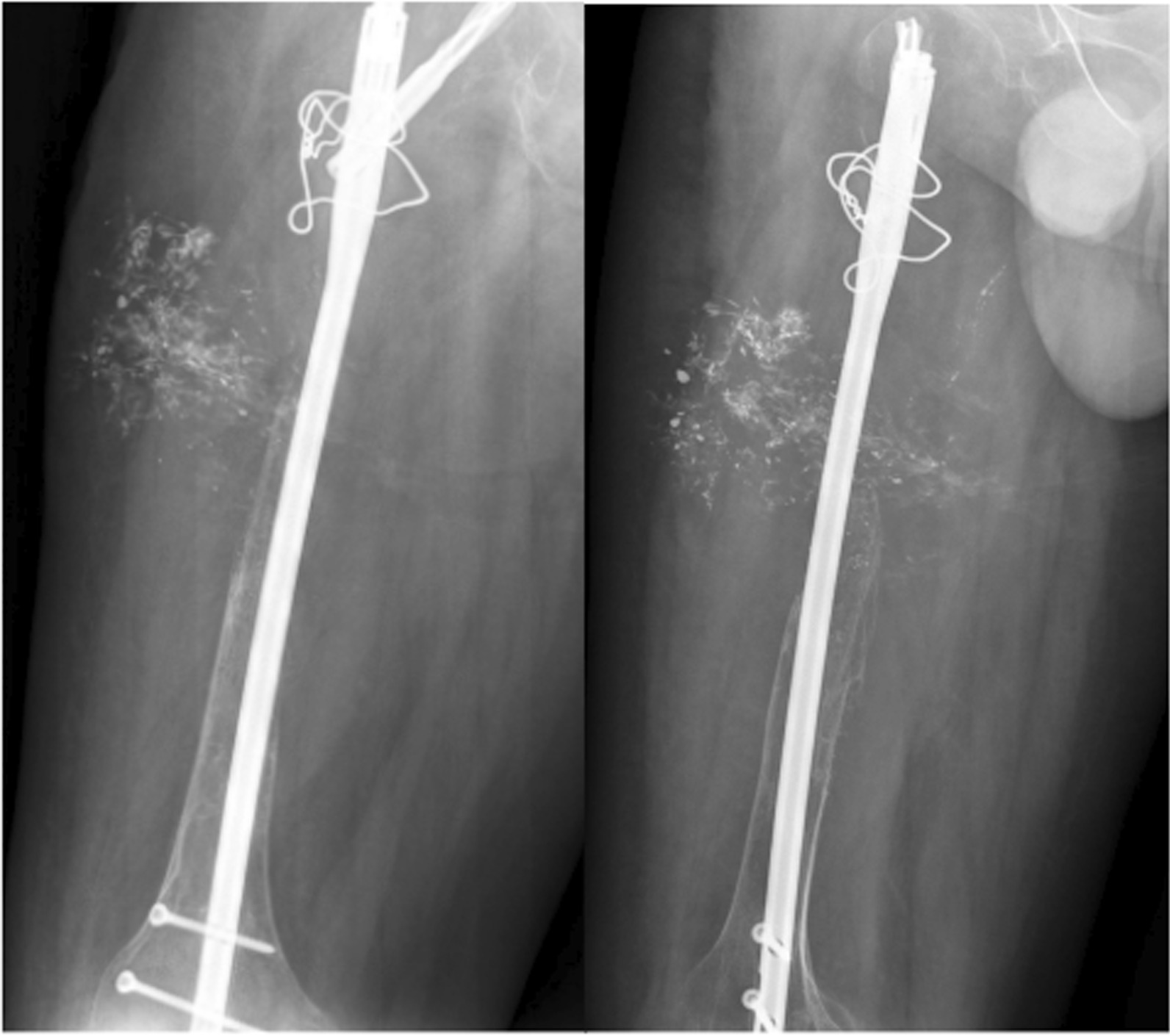
Right femoral X-ray of patient when he was referred to us

After approximately 18 months following the initial biopsy, the patient was referred to our center (Fig. 5). There was no improvement in pain and the disease had progressed. Upon visiting our center, the patient had been experiencing severe pain and was wheelchair-bound and completely disabled. Skin examination revealed no abnormalities except for surgical incisions, and joint movements were limited due to pain. Laboratory tests such as inflammation tests and bone metabolic tests were normal.

**Fig. 6:**
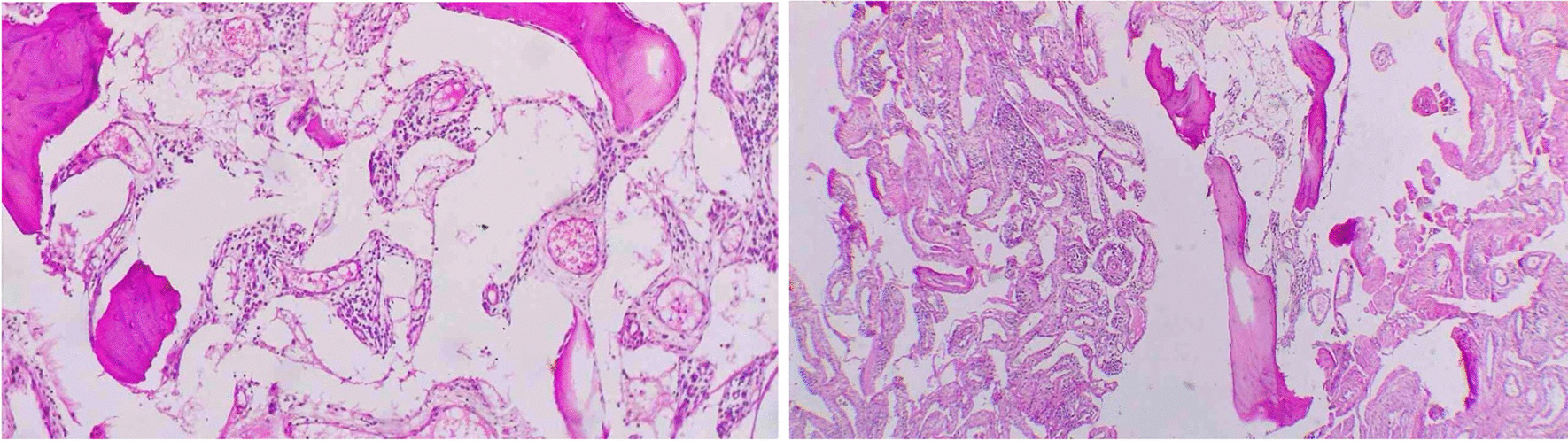
Microscopic examination showing thin bone trabeculae and intervening spaces accompanied with significant proliferation of lymphatic and vascular channels; infiltration of chronic inflammatory cells including lymphocytes and plasma cells is conspicuous; low magnification, hematoxylin and eosin stain

After considering the patient’s age, laboratory test results, and the presence of lymphatic and vascular channel proliferation along with bone trabeculae, as well as the absence of cellular atypia in the histological review (Fig. 6) and other accompanying symptoms, a multidisciplinary team arrived at the conclusive diagnosis of Gorham–Stout disease (GSD). While non-surgical treatments are typically the preferred course of action for patients with GSD, the severity of the patient’s pain and impaired mobility necessitated a multidisciplinary approach to his care. After careful consideration and evaluation, the decision was made to proceed with surgical intervention. The surgical plan entailed a wide resection of the lesion and reconstruction utilizing a MUTARS® femoral system endoprosthesis.

## Surgical technique

**Fig. 7:**
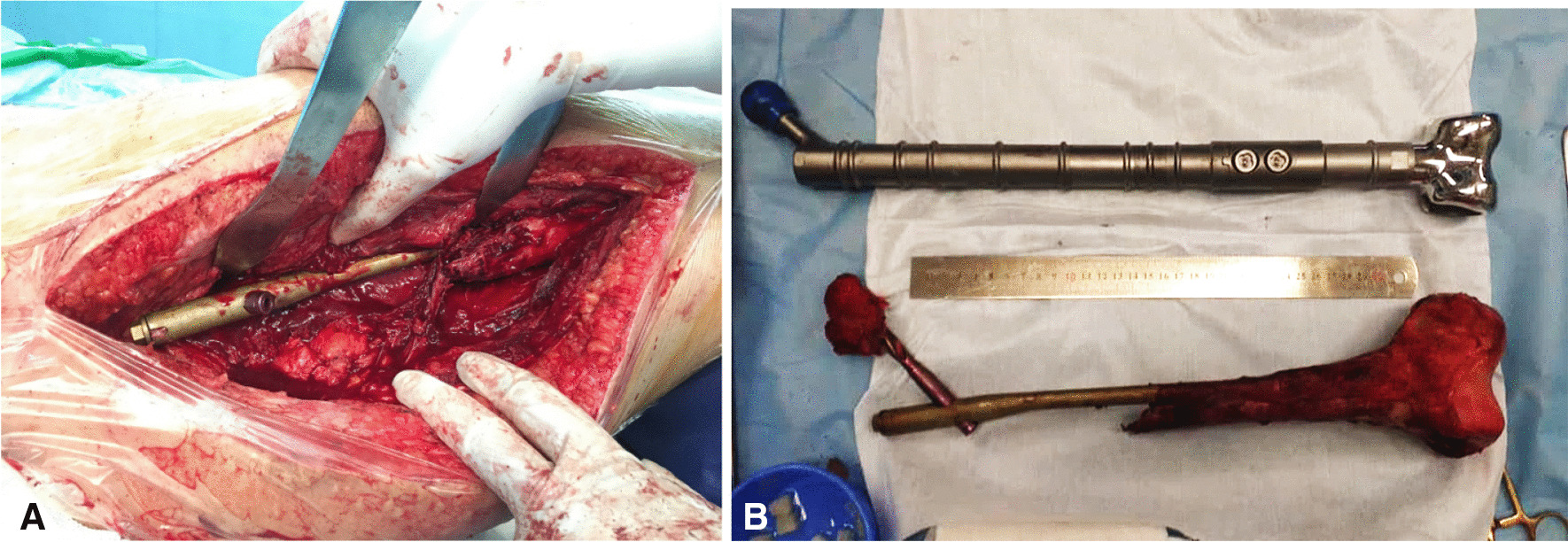
Extended lateral approach (**A**) and entire resection piece (**B**)

Following general anesthesia, the patient was positioned supine with a pillow placed under their right buttock. After making an incision through the skin, subcutaneous tissue, and fascia, a lateral approach was used to expose the bone site. Despite encountering healthy muscles and soft tissues proximally, we found an absence of bone tissue in this region, with the cephalomedullary nail located between the muscles and a thin shell of hard tissue inside the joint. Distally, we observed the presence of bone tissue, but the bone was extremely fragile and soft. Given these findings, we decided to proceed with a total femur replacement and hip joint replacement (Fig. 7).

**Fig. 8:**
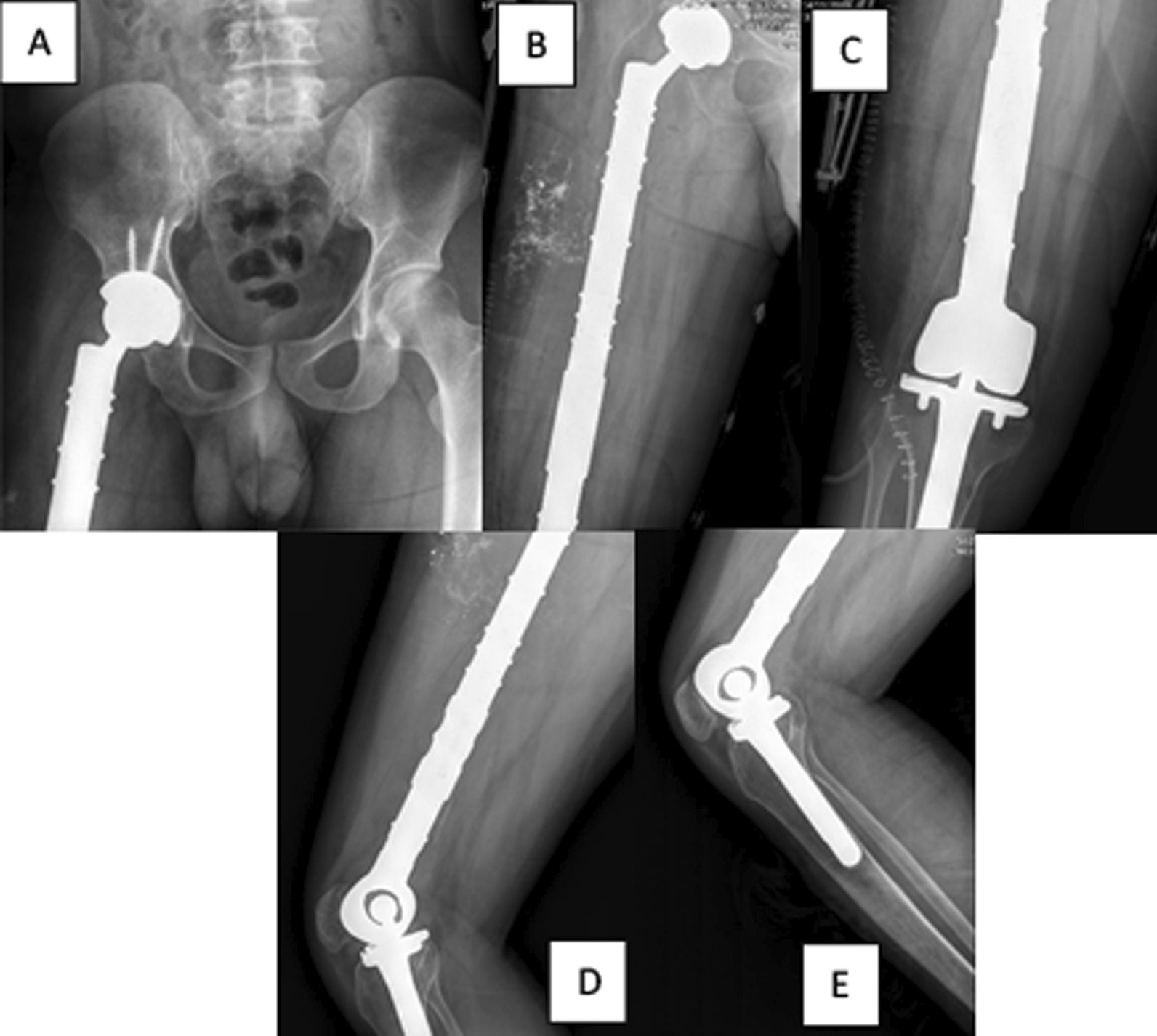
Postoperative radiograph including Anteroposterior view of pelvic (**A**), femur (**B**), knee (**C**), and lateral view of the femur (**D**) and knee (**E**)

The prosthetic limb was inserted and adjusted to the appropriate length and rotation, after which we reconstructed the ligamentous muscle connections. Following hemostasis and drain installation, we closed the wound layer by layer (Fig. 8).

**Fig. 9:**
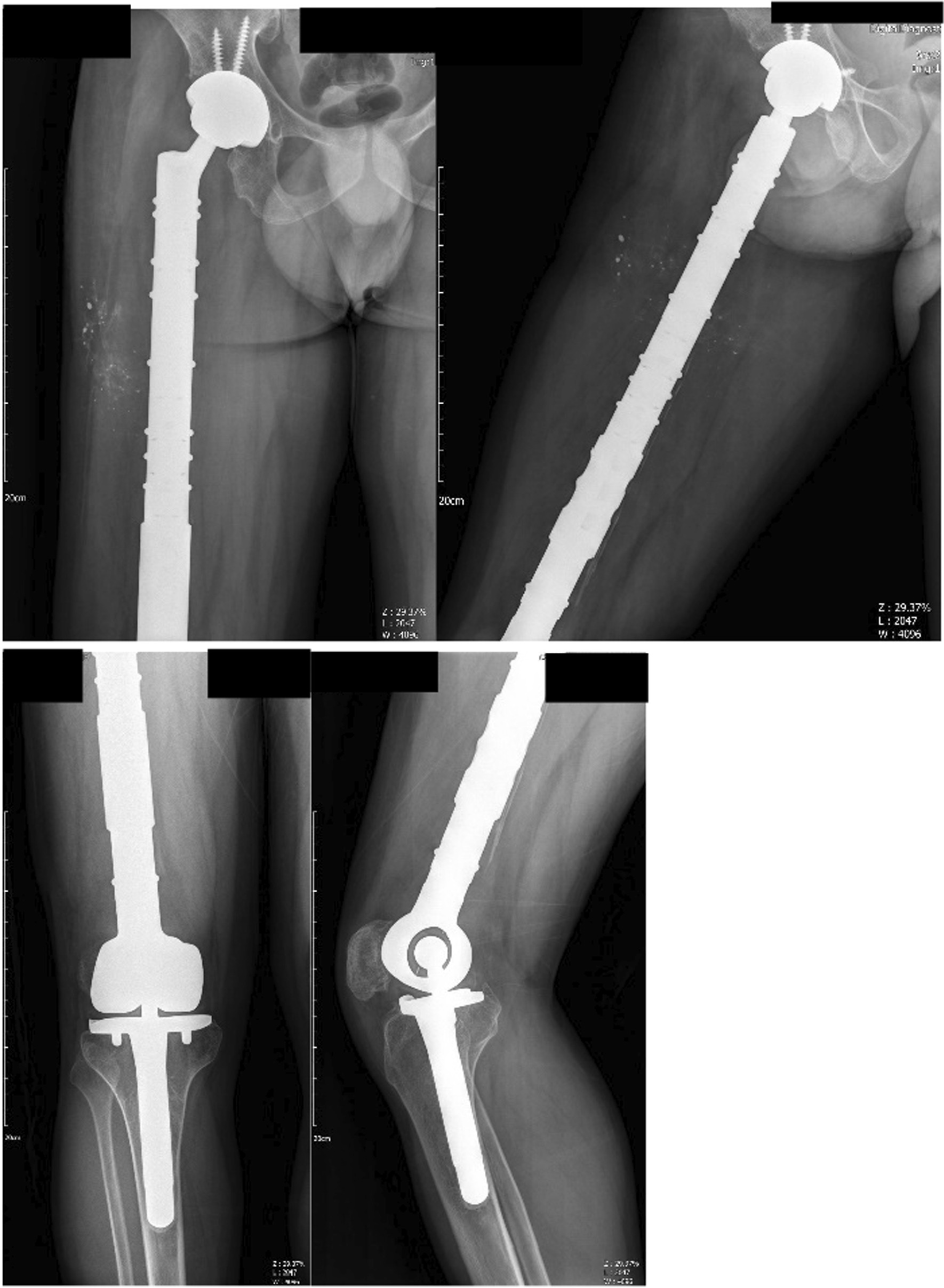
X-ray images 5 years postoperative

The patient underwent an abduction brace for 10 weeks and a physiotherapy program for 14 months without additional medical treatment. After 5 years of follow-up, the patient can walk independently with a slight limp and without pain (Fig. 9).

## Discussion and conclusion

Gorham–Stout disease (GSD) is a rare disease, with fewer than 400 cases reported in the medical literature to date [5]. It typically presents in the second and third decades of life, although cases outside this age range have been reported. Gender-based differences in the prevalence of GSD have been noted in most studies as equal [6, 7], but limited reports indicate a higher incidence in men [8, 9].

The precise pathophysiology of GSD remains poorly understood, although cellular and molecular studies have shed some light on potential mechanisms [10, 11]. These studies suggest that the disease is characterized by an imbalance in bone metabolism, with stimulated bone resorption and a lack of replacement leading to the replacement of bone tissue by fibrotic tissue. While most cases involve a single affected area, multiple sites of involvement have been reported, particularly in the spine and ribs [12]. The most commonly affected regions include the spine, ribs, mandible, shoulder, and pelvic girdle [13, 14].

Diagnosing GSD requires ruling out other potential causes, as it is “a diagnosis of exclusion” [15]. We observed large cystically dilated vessels with thin walls in the patient’s histological examination. However, we did not see the presence of blowing bone lacunae with high ossification and specific organization, such as central microcysts and perpendicular spicules at the surface. These features are typically observed in bone hemangiomas [16].

The differential diagnosis of spontaneous and progressive osteolysis may include several diseases such as Gorham–Stout disease, Paget’s disease of bone, melorheostosis, multicentric carpotarsal osteolysis syndrome (MCTO), and Torg syndrome. The final diagnosis for the patient was determined on the basis of several factors including age, laboratory test results, the presence of proliferation of lymphatic and vascular channels along with bone trabeculae in the pathological view, and the absence of accompanying symptoms.

Despite the patient’s normal bone metabolic tests, blood tests, and inflammatory tests, no other diagnosis could explain the disease’s progression to disability. Ultimately, the patient was diagnosed at our center, a referral center for musculoskeletal tumors, through multidisciplinary team collaboration.

Heffez *et al*. developed a set of diagnostic criteria for GSD, which includes: (1) biopsy showing angiomatous or fibrous connective tissue without cellular atypia, (2) minimal or no osteoblastic response or dystrophic calcifications, (3) evidence of progressive bone resorption, (4) non-ulcerative and non-cortical-expanding lesions, (5) no visceral involvement, (6) osteolytic radiographic pattern, and (7) negative hereditary, metabolic, neoplastic, immunological, and infectious etiology [17].

To reach the diagnosis of GSD, a high degree of clinical suspicion and collaboration between various specialists is essential. In this patient’s case, while it may have initially appeared typical in retrospect, the presentation was atypical, underscoring the importance of careful evaluation and thorough exclusion of other potential diagnoses.

Treatment for GSD typically involves medical therapy, with calcium and vitamin D supplements, bisphosphonates, sirolimus, and interferon being some of the key drug categories used [5, 18]. Rossi recommended including agents that prevent bone resorption and inhibit angiogenesis and anabolic agents in treatment [10]. However, the prediction of disease progression remains uncertain [19], and patients may experience new symptoms and progression even after years of treatment [20], However, other reports have not shown recurrence of the disease [14, 18].

Another treatment option for GSD is radiotherapy, which is recommended as an adjuvant treatment and has been reported to be effective in up to 80% of cases, according to a report by Heyd *et al*. [21]. However, radiotherapy is not currently recommended as a primary treatment and is typically used in conjunction with surgical treatment if medical therapy is not effective [5]. In the case of our patient, adjuvant radiotherapy was not performed as the entire femur bone was affected.

There is no interest in performing curettage and excision surgery for GSD, with many surgeons opting for lesion complete resection instead. Resection amounts are determined on the basis of imaging modalities, although there are concerns that any remaining bone may begin to undergo resorption over time. While some articles have reported success with allograft reconstructions [22], others have raised concerns about resorption [7, 23]. In our case, a structural graft was used but absorbed quickly. Few articles have discussed using prosthetic implants, whether cemented or non-cemented [24–26]. However, in our patient, the unfavorable condition of the remaining femur shaft necessitated a total femur replacement, which was ultimately performed. Notably, the patient’s soft tissue remained intact, and proper reconstruction was carried out to minimize lameness and performance limitations.

In conclusion, diagnosing and treating GSD pose significant challenges to physicians. Our case report highlights GSD’s clinical presentation and management difficulties, specifically in the case of entire femur involvement. Despite attempts at appropriate treatments, the lack of a clear diagnosis prolonged the patient’s procedure and delayed the initiation of medical treatment. Ultimately, endoprosthesis surgery was deemed necessary. We suggest that the presence of osteolysis without a clear tumoral pathology but with vascular abnormalities should raise suspicion for GSD. Early introduction of treatment is strongly recommended to prevent bone resorption and promote complete healing. However, it should be noted that disease progression cannot be predicted.

## Data Availability

The datasets generated during and/or analyzed during the current study are available from the corresponding author on reasonable request. The materials used in this study are available commercially or can be obtained from the authors upon request.

## Abbreviations

GSD: Gorham–Stout disease
MUTARS®: Modular Universal Tumour and Revision System
